# Validation and identification of anoikis-related lncRNA signatures for improving prognosis in clear cell renal cell carcinoma

**DOI:** 10.18632/aging.205568

**Published:** 2024-02-21

**Authors:** Zhenjie Zhu, Qibo Wang, Xiaowei Zeng, Shaoxing Zhu, Jinchao Chen

**Affiliations:** 1 Zhejiang Cancer Hospital, Institute of Basic Medicine and Cancer (IBMC), Chinese Academy of Sciences, Hangzhou, China; 2 Fujian Medical University Union Hospital, Fuzhou, China

**Keywords:** clear cell renal cell carcinoma, anoikis-related lncRNAs, risk model, immune infiltration landscape, prognostic value

## Abstract

Background: Clear cell carcinoma (ccRCC) usually has a high metastasis rate and high mortality rate. To enable precise risk stratification, there is a need for novel biomarkers. As one form of apoptosis, anoikis results from the disruption of cell-cell connection or cell-ECM attachment. However, the impact of anoikis-related lncRNAs on ccRCC has not yet received adequate attention.

Methods: The study utilized univariate Cox regression analysis in order to identify the overall survival (OS) associated anoikis-related lncRNAs (ARLs), followed by the LASSO algorithm for selection. On this basis, a risk model was subsequently established using five anoikis-related lncRNAs. To dig the inner molecular mechanism, KEGG, GO, and GSVA analyses were conducted. Additionally, the immune infiltration landscape was estimated using the ESTIMATE, CIBERSORT, and ssGSEA algorithms.

Results: The study constructed a novel risk model based on five ARLs (AC092611.2, AC027601.2, AC103809.1, AL133215.2, and AL162586.1). Patients categorized as low-risk exhibited significantly better OS. Notably, the study observed marked different immune infiltration landscapes and drug sensitivity by risk stratification. Additionally, the study preliminarily explored potential signal pathways associated with risk stratification.

Conclusion: The study exhibited the crucial role of ARLs in the carcinogenesis of ccRCC, potentially through differential immune infiltration. Furthermore, the established risk model could serve as a valuable stratification factor for predicting OS prognosis.

## INTRODUCTION

In 2018, about 403,000 cases of renal cancer were newly diagnosed worldwide, bringing huge challenges to public health and serious socio-economic burden [1, 2]. As a heterogeneous tumor, clear cell carcinoma (ccRCC) accounts for 80% of renal cell carcinoma [3]. Different from advanced ccRCC, patients with early diagnosis and treatment have a better prognosis [2]. Although many cancer biomarkers have been discovered, their accuracy in predicting the prognosis of patients has not been clinically recognized and applied. The mining of biomarkers can improve the efficiency of diagnosis and the efficacy of cancer treatment.

Anoikis is one apoptosis form due to disruption of cell-ECM attachment or cell-cell connection [4]. Under physiological conditions, anoikis eliminates misplaced or shed cells and contributes to tissue homeostasis. Under pathological conditions, anoikis is involved many pathological processes including tumorigenesis. By dissociating from the ECM and avoiding systemic apoptosis in the process, cancer cells eventually spread, thus loss of opportunity for surgery [4]. This process involves genetic and molecular changes that allow cells to survive without ECM attachment [5]. Recent studies have shown that anoikis has potential therapeutic value in RCC. Some anoikis key genes was reported to promote proliferation and migration in RCC [6]. In addition, progression of RCC can be inhibited by reversing anoikis resistance [7]. Quinazolines activates anoikis by AKT signal pathway adjustment, leading to an antitumor effect [8]. Anoikis resistance is one key characteristic of metastatic tumor cells. However, as a tumor prone to hematogenous metastasis, the mechanism of anoikis in ccRCC is not fully studied. Multiple biological functions of ccRCC are regulated by ECM by providing adhesion substrates and modulating signal transduction, including proliferation, angiogenesis and invasion [9]. There is evidence that increased anoikis activity can reduce the invasive ability of ccRCC cancer cells through regulation of key protein expression levels [10]. All these evidences indicate the potential role of anoikis in ccRCC.

The immune microenvironment (TME) plays a critical role in ccRCC carcinogenesis. The dynamic and complex role of TME includes both immunostimulation and immunosuppression [11]. Tumor-infiltrating lymphocytes (TILs) are associated with a favorable prognosis for ccRCC. The antitumor immune-response can be inhibited by the high density of myeloid-derived suppressor cells (MDSC) in ccRCC [12]. In addition, tumor-associated macrophages (TAM) as well as cancer-associated fibroblasts (CAF) also participated in the shaping of TME [13]. Due to the important role of TME, the treatment regimen that targets TME-related groups also has positive clinical value [14]. Immuno-related therapies have achieved initial efficacy in clinical trials of ccRCC [13]. Further exploration of immune-related pathways and related targets is of great significance.

Long non-coding RNA (lncRNA), as a type of RNA that exists in the nucleus or cytoplasm, has a transcript length of more than 200nt [15]. LncRNAs have been shown to be involved in many important gene expression regulation processes, including chromatin modification, transcriptional interference, DNA methylation and histone modification [16]. Increasing evidence showed in the development of RCC, lncRNAs interact with a variety of RNAs and proteins at transcription, post- transcription and epigenetic level, which further leads to the involvement of RCC invasion and metastasis [17–20]. According to the 2021 review, anoikis-related lncRNAs including ANRIL, FOXD2-AS1, HOTAIR, and SNHG12 have been unveiled to participate in the process of tumor metastasis, stem cell formation and tumor survival [4]. In addition, the combination of chemotherapy drugs and specific lncRNAs can improve the therapeutic effect [21, 22]. Therefore, risk stratification based on lncRNA has a clinical value. However, the research of anoikis-related lncRNAs in ccRCC in insufficient.

To dig the role of anoikis-related lncRNAs (ARLs) in ccRCC, we built a novel risk signature based on 5 ARLs and verified its effectiveness in predicting the prognosis of patients with ccRCC. Immune microenvironment differences by risk stratification were comprehensively analyzed. Differences in immune response and drug sensitivity by risk stratification were also further explored. In view of the rapid development of targeted drugs in ccRCC treatment [23], our study provides idea for further finding the ideal target responsible for disease development and clinical application potential.

## MATERIALS AND METHODS

### Data download

The open transcriptome matrix and clinicopathological characteristic information were collected from TCGA. After excluding samples with no effective survival time, 525 samples were included for analysis. The gene expression matrix was extracted by Perl scripts. The ensemble human genome browser GRCh38. p13 was utilized to annotate the symbol of mRNA and lncRNA.

### Identification of anoikis-related lncRNAs (ARLs)

From the MSigDB database, 34 anoikis-related genes (ARGs) were obtained (Supplementary Table 1). Pearson correlation analysis was set at a threshold of |correlation coefficient (r) | > 0.6, and *P* < 0.001 [24]. Then, 44 lncRNAs were identified as ARLs for analysis (Supplementary Table 2).

### ARLs risk model establishment

On the basis of univariate-LASSO Cox algorithm to identify the overall survival (OS) related ARLs, the risk score = (0.683 x AL162586.1) + (-0.753 x AC027601.2) + (-0.993 x AC103809.1) + (1.679 x AL133215.2) + (-0.414 x AC092611.2). The median of risk score is used to stratify risk. The Kaplan-Meier survival curve was utilized by R package “survival”. The training and test cohort were randomly divided with a ratio of 7:3 [25].

### Molecular functional evaluation

The differently expressed genes (DEGs) of ccRCC by risk stratification were identified by algorithm “limma” (|FC| ≥ 2, *P* < 0.05). Metascape database was used to perform DEGs enrichment analysis. The GO analysis and KEGG analysis were estimated via “clusterProfiler” R package [26].

### Independent prognosis analysis

Univariate/multivariate Cox analysis were carried out via R package “survival”. Algorithm “timeROC” was conducted to evaluate the AUC at 1-, 3-, and 5-years. A nomogram model was constructed via “rms” script. The consistence of OS rate predicted nomogram and actual OS rate was evaluated by calibration diagram.

### Consensus clustering analysis of ccRCC

Different molecular subtype clustering from ccRCC samples was done via algorithm “ConsensusClusterPlus” by R language based on the 5 prognostic ARLs. Partitioning around medoids with “euclidean” distances and optimal classification of K = 2-9 were used for clustering process.

### Immune microenvironment landscape and drug sensitivity evaluation

The estimate scores were estimated by algorithm “estimate” from R package. CIBERSORT and ssGSEA algorithms from R package were employed to calculate the immune cell proportions. “GSVA” script was used to estimate the immune function score. Immunophenoscore (IPS) results, Immune Dysfunction and Exclusion (TIDE) score and drug sensitivity data were obtained from TCIA database, TIDE database and GDSC database, respectively.

### Statistical analysis

All statistical analysis were performed by using the R software (version 4.1.0) and Perl scripts. Wilcoxon rank-sum test and ANOVA were used for the assessment differential functions of two groups and multiple groups, respectively. All statistical significance were set at *P* < 0.05.

## RESULTS

### Generation of prognostic anoikis-related lncRNAs (ARLs)

**Figure 1:**
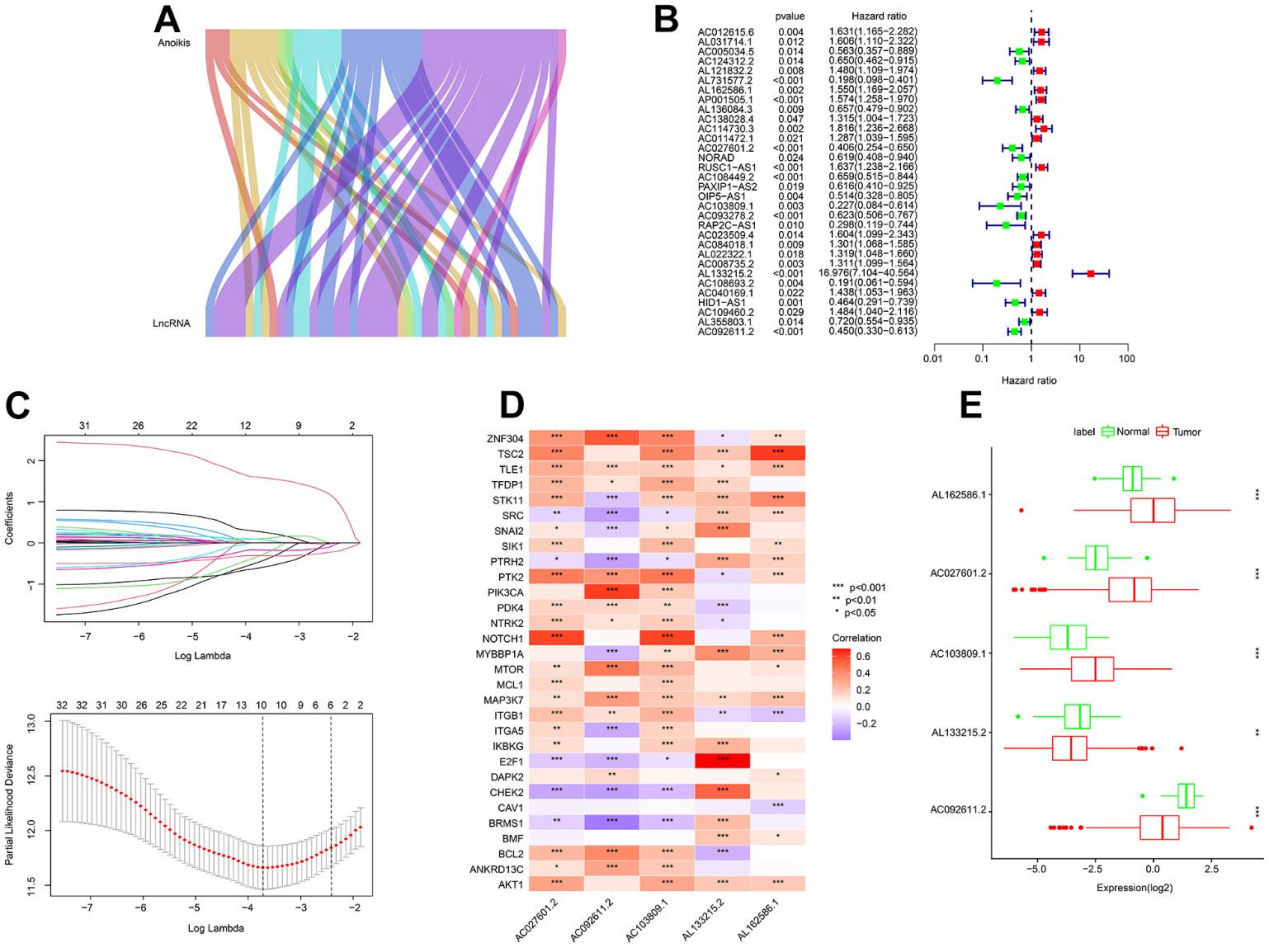
**Identification of prognostic ARLs.** (**A**) Identification of lncRNAs associated with anoikis-related genes by the Sankey diagram. (**B**, **C**) Prognostic ARLs identification via univariate-LASSO analysis. (**D**) Correlation analysis of the prognostic ARLs and ARGs. (**E**) The expression profiler of prognostic ARLs in the normal and tumor groups.

As shown in Sankey plot, a total of 44 lncRNAs associated with anoikis-related genes were identified as anoikis-related lncRNAs (ARLs) in this study (Figure 1A and Supplementary Figure 1). By the univariate-LASSO analysis, 10 OS associated ARLs for ccRCC were identified (Figure 1B, 1C). 5 prognostic ARLs were confirmed by multivariate Cox regression analysis to be able to independently predict the OS rate, which further led to the establishment of the ARLs risk model. Correlation analysis results suggested a significant association between the 5 selected ARLs and anoikis-related genes (Figure 1D). As shown in Figure 1E, the expressions of AL162586.1, AC027601.2, and AC103809.1 were relatively highly expressed in the tumor group, while the expressions of AL133215.2 and AC092611.2 were higher in the normal group.

### Construction of ARLs risk model in ccRCC

**Figure 2:**
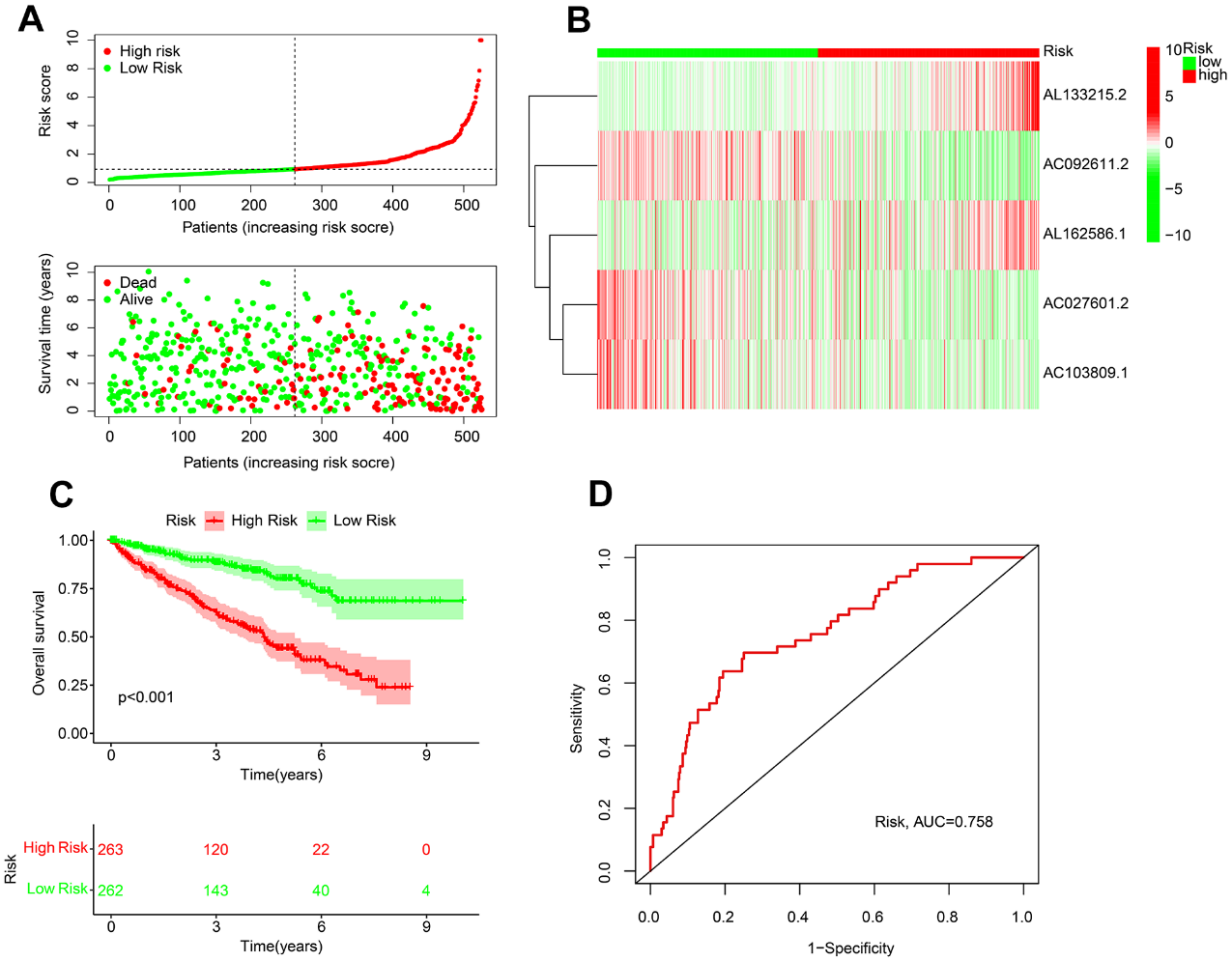
**Risk model establishment of the prognostic ARLs in ccRCC.** (**A**) Distribution plot of the risk score and correlation analysis of the survival time and risk score of ccRCC patients. (**B**) The expression of prognostic ARLs in ARLs score subgroup. (**C**) Kaplan-Meier survival curve of ccRCC patients in ARLs score subgroup. (**D**) ROC curve of risk model, and the AUC was 0.756.

Since 5 ARLs with ccRCC prognostic value were selected, a novel risk model was further established. Patients with ccRCC were risk stratified according to their ARLs risk scores. The inverse association between the survival time and risk score could be observed (Figure 2A). Heatmap diagram showed the expressions of AC092611.2, AC027601.2, and AC103809.1 were overexpressed in the low-risk group, whereas the expressions of AL133215.2 and AL162586.1 were higher in the other group (Figure 2B). The results of KM survival curve suggested a large advantage in survival for patients for patients with low risk (Figure 2C). The ROC curve results show an AUC value of 0.758 for this novel ARLs risk model (Figure 2D).

### Validation of ARLs prognostic signature in ccRCC

**Figure 3:**
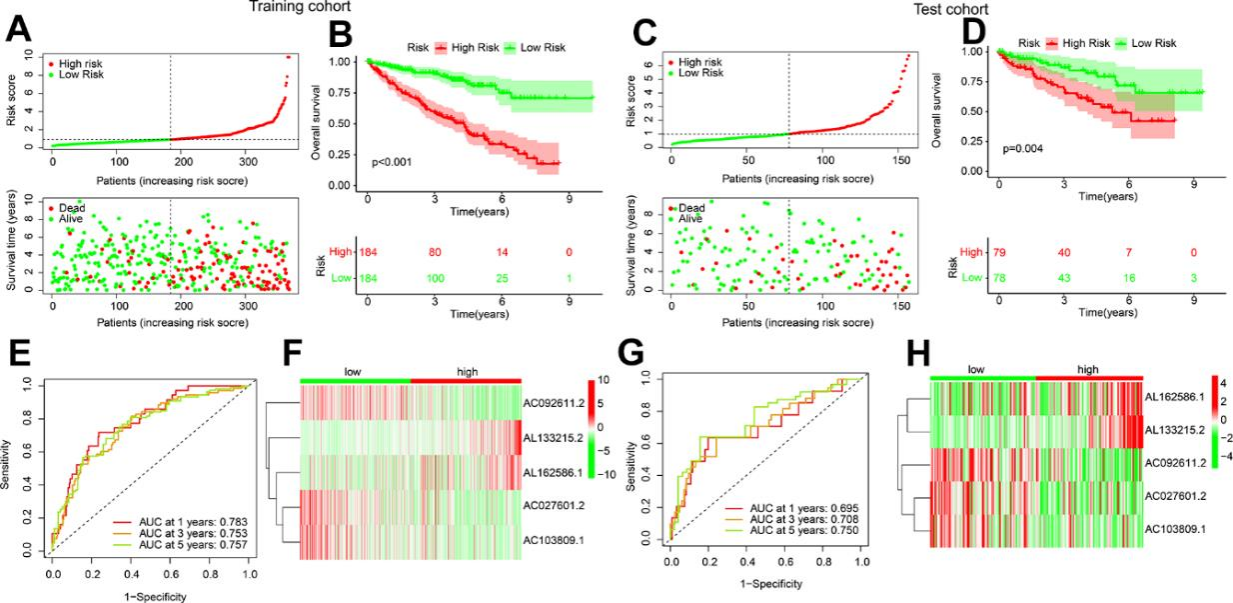
**Validation of risk model in training cohort and test cohort.** (**A**) Distribution of the risk score and correlation analysis of survival time and risk score in training cohort. (**B**) Kaplan-Meier survival curve of ccRCC patients with in the low- and high-risk group in training cohort. (**C**) Distribution of the risk score and correlation analysis of survival time and risk score in test cohort. (**D**) Kaplan-Meier survival curve of ccRCC patients with in the low- and high-risk group in test cohort. (**E**–**H**) ROC curve analysis and the expression of 5 ARLs in low- and high-risk group in training and test cohort.

An internal validation was subsequently developed to investigate the independence and accuracy of the established ARLs risk model in prognosis evaluating. The training and test cohorts were randomly divided into 525 ccRCC patients in a 7:3 ratio. Patients in the training and test cohorts were then randomly divided into two subgroups. Survival time was demonstrated by scatter plots to be negatively correlated to risk score in both groups (Figure 3A, 3C). In both cohorts, significantly better OS rate could be observed in the low-risk group, which was shown by Kaplan-Meier analysis (Figure 3B, 3D). The results of time ROC curve exhibited the acceptable AUC values at 1-, 3-, and 5-years for both training and test cohort (Figure 3E, 3G). The heatmap diagrams showed that the expressions of AC092611.2, AC027601.2, and AC103809.1 were higher, while the expression of AL133215.2 and AL162586.1 were lower in the low-risk group (Figure 3F, 3H). These results indicate the effectiveness of the established ARLs in predicting the ccRCC prognosis.

### Risk scores for different clinicopathological features

**Figure 4:**
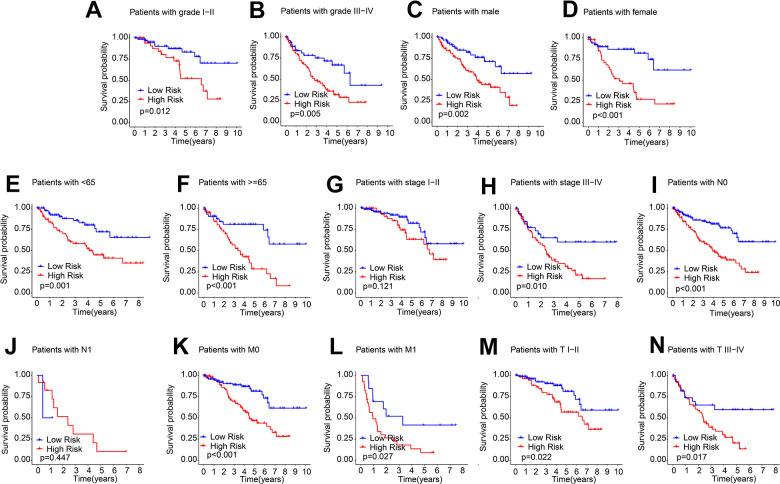
**Prognostic analysis of ARLs score in different clinical features.** The prognostic KM curve of ccRCC samples among the (**A**) Grade I-II; (**B**) Grade III-IV; (**C**) Male; (**D**) Female; (**E**) Age < 65; (**F**) Age ≥ 65; (**G**) Stage I-II; (**H**) Stage III-IV; (**I**) N0; (**J**) N1; (**K**) M0; (**L**) M1; (**M**) T I-II; (**N**) T III-IV.

The subgroup analysis was subsequently performed. According to the median risk score, patients were stratified according to different clinicopathological features by dichotomy. The OS rate of ccRCC patients in the low-risk group were significantly higher in grade I-II, grade III-IV, male, female, age < 65, age ≥ 65, stage III-IV, N 0, M 0, M 1, T I-II, and T III-IV. However, the OS rate of patients with ccRCC in stage I-II and N 1 was similar (Figure 4). These results illustrate the established risk score allows prognosis assessment of different clinical features.

### Independence analysis of ARLs-based prognostic model of ccRCC

**Figure 5:**
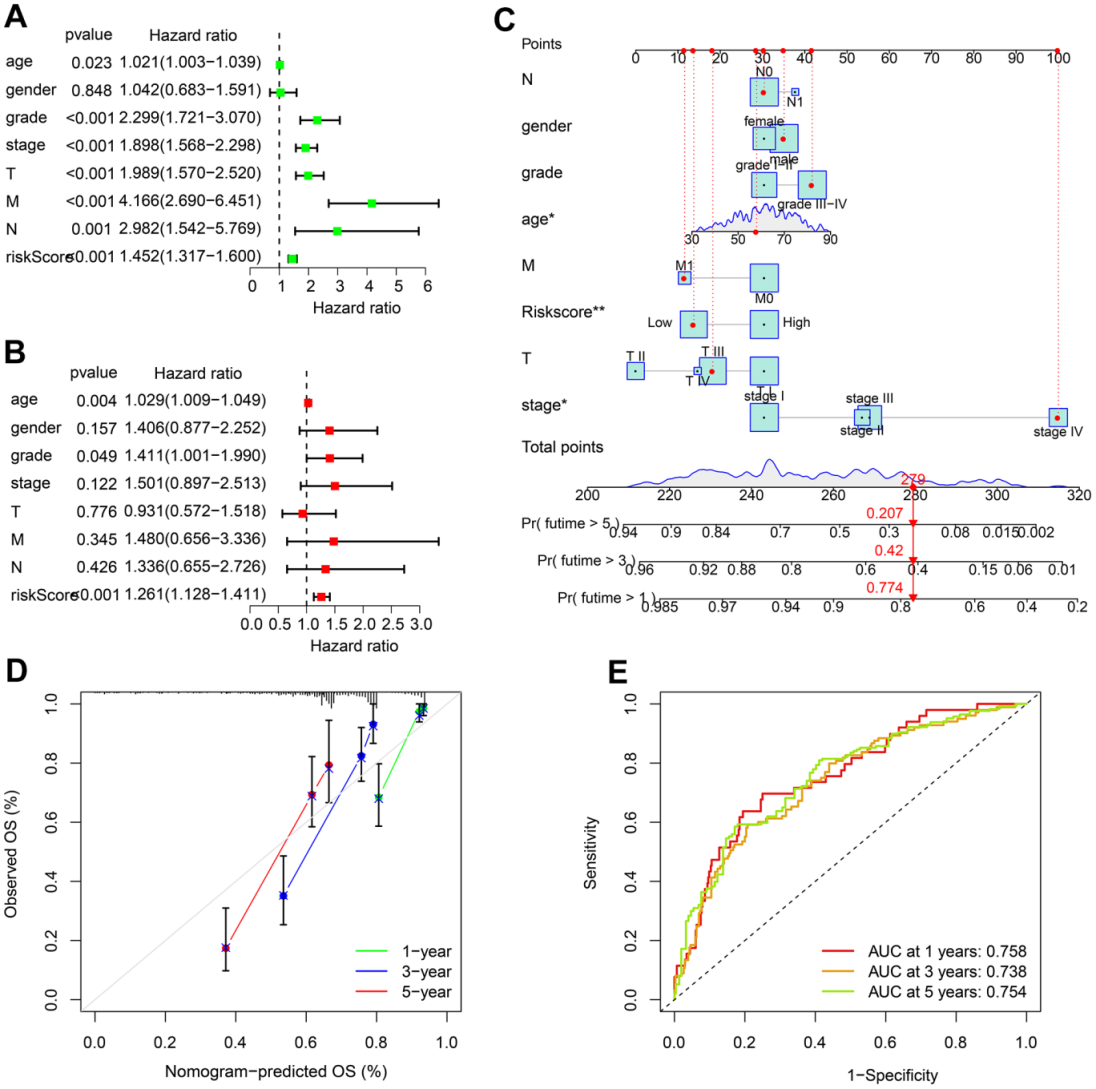
**Independence evaluation of ARLs risk model.** (**A**) Univariate and (**B**) multivariate analysis reveals the independence of ARLs score and clinical features of ccRCC. (**C**) Nomogram model constructed based on the ARLs risk model and clinicopathological characteristics. (**D**) Calibration curve shows the consistence of the OS rate predicted by nomogram. (**E**) Time-dependent ROC curve.

Univariate Cox regression analysis showed in addition to multiple clinical characteristics, risk score was also significantly correlated with ccRCC OS rate (Figure 5A). Multivariate Cox regression analysis further demonstrated risk score as an independent prognostic indicator for ccRCC patients (Figure 5B). We subsequently established nomograms to accurately predict patient survival time (Figure 5C). Calibration curves showed that the OS rates predicted by the nomogram were consistent with the actual OS rates (Figure 5D). The time dependent ROC curve also yielded acceptable AUC values for 1, 3, and 5 years (Figure 5E). In summary, the independent prognostic significance of ARLs risk model for ccRCC prognosis was confirmed.

### Molecular function analysis of ARLs-related risk groups

**Figure 6:**
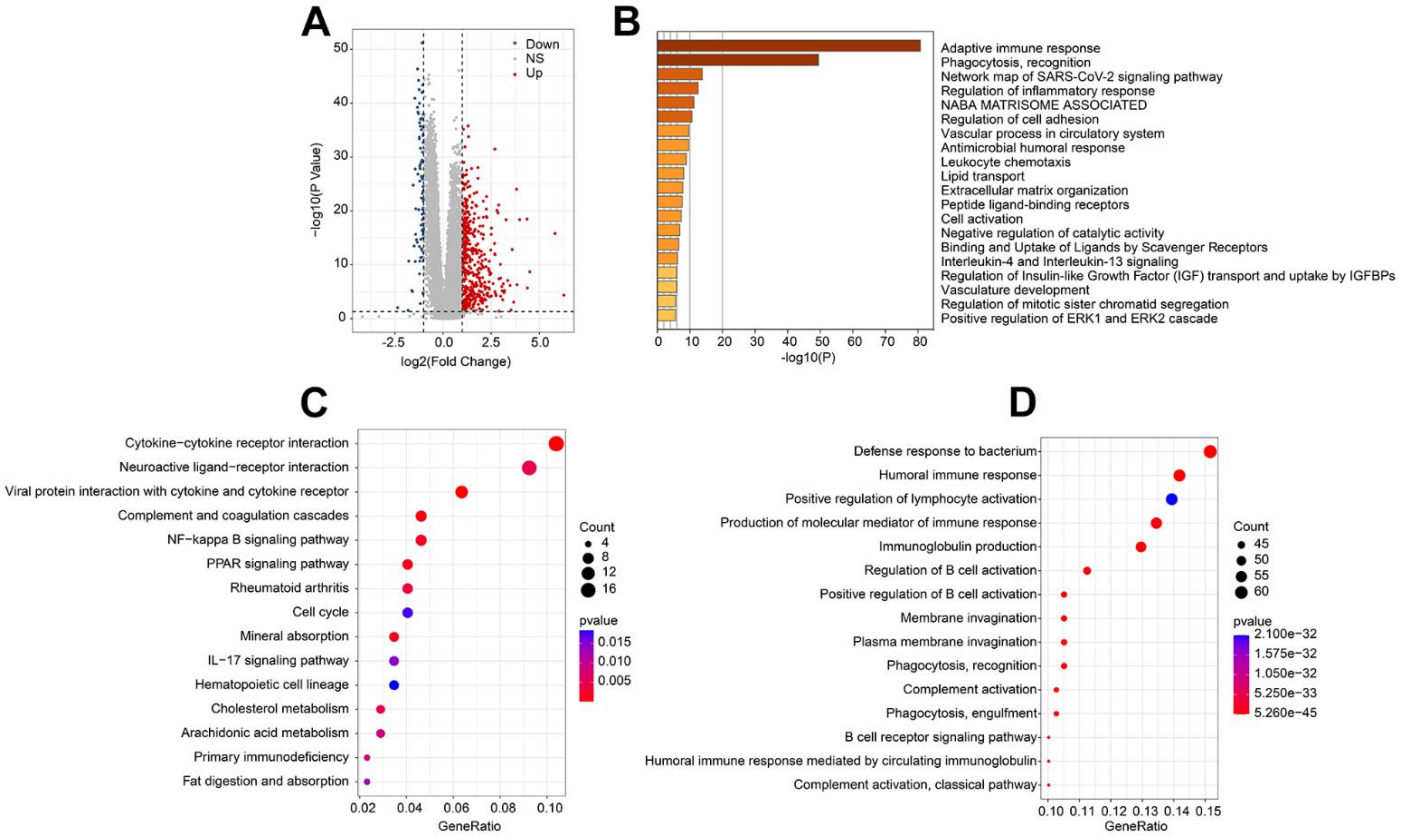
**Molecular functional analysis of ARLs score subgroups.** (**A**) Generation of the DEGs of ARLs score subgroups. (**B**) Enrichment analysis of DEGs. (**C**) KEGG and (**D**) GO enrichment analysis of ARLs score subgroups.

To investigate the potential molecular mechanism of ARLs risk model, multiple enrichment analysis algorithms were further performed. Volcano diagram illustrated the DEGs by risk stratification, showing in the high-risk group, most of the DEGs were significantly upregulated (Figure 6A). Subsequently performed enrichment analysis result suggested the DEGs were significantly enriched in some immune related signaling pathways, such as adaptive immune response and phagocytosis, recognition (Figure 6B). KEGG enrichment analysis illustrated that cytokine−cytokine receptor interaction was enriched (Figure 6C). Enriched immune related biological processes could be observed by GO enrichment analysis, including defense response to bacterium, positive regulation of lymphocyte activation and humoral immune response (Figure 6D). These results suggested the role of immune-associated signaling pathways in mediating ARL-related functions.

### Characteristic of molecular subtypes and immune microenvironment analysis

**Figure 7:**
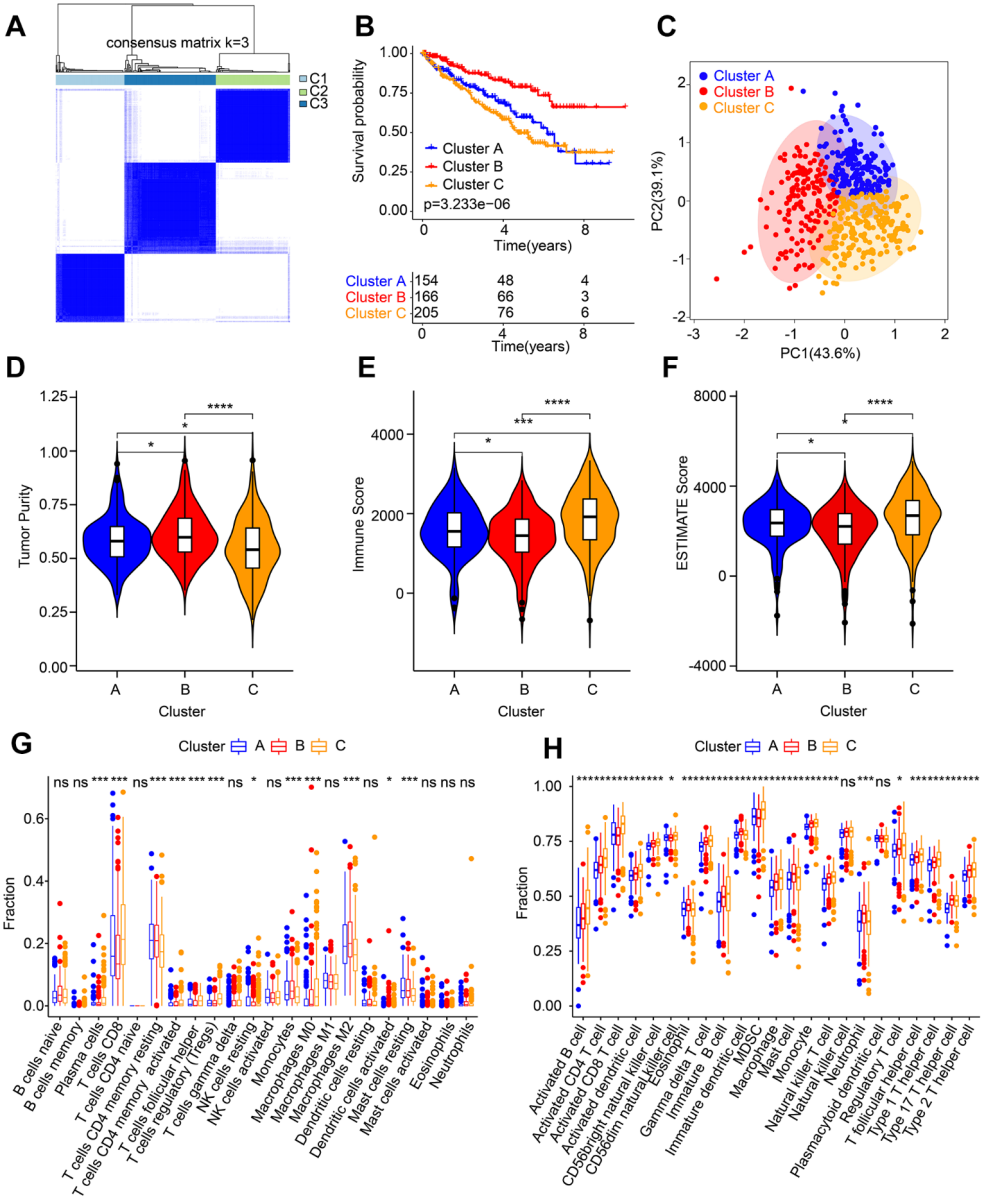
**Molecular subtypes analysis and immune microenvironment landscape characterization.** (**A**) Identification of the molecular subtypes for ccRCC. (**B**) Clinical prognostic analysis of Cluster A, Cluster B and Cluster C. (**C**) PCA score plot shows a significant distribution pattern of patients in Cluster A, Cluster B and Cluster C. (**D**–**F**) Tumor purity, immune and ESTIMATE scores. The proportion of immune cells calculated by (**G**) CIBERSORT and (**H**) ssGSEA.

Based on the selected 5 ARLs, consensus clustering was performed for ccRCC subtype clustering. The heat map shows the optimal classification when K = 3, with classes A, B and C having 154, 166 and 205 samples respectively (Figure 7A). Survival curve exhibited that class B patients had the most optimistic OS rate (Figure 7B). The principal component analysis score plot illustrated a clear separation of among three groups (Figure 7C). ESTIMATE algorithm results suggested that Cluster B patients had higher tumor purity than the other two groups; however, the immune and ESITMATE score were lower (Figure 7D–7F).

In addition, more efforts were made to estimate the component of immune cells in the different subtypes. The result of CIBERSORT showed that Cluster C patients had higher infiltration level of plasma cells, CD8^+^ T cells, follicular helper T cells, regulatory T cells (Tregs) and M0 macrophages, whereas the infiltration level of resting CD4^+^ memory T cells, monocytes, resting NK cells, and M2 macrophages were lower (Figure 7G). ssGSEA algorithm result suggested Cluster C had significantly higher portions of immune cells in comparison to those in Cluster A and B (Figure 7H). These findings demonstrate that different ccRCC molecular subtypes were closely related to patient prognosis as well as immune infiltration.

### Association of ARLs risk model and immune microenvironment landscape

**Figure 8:**
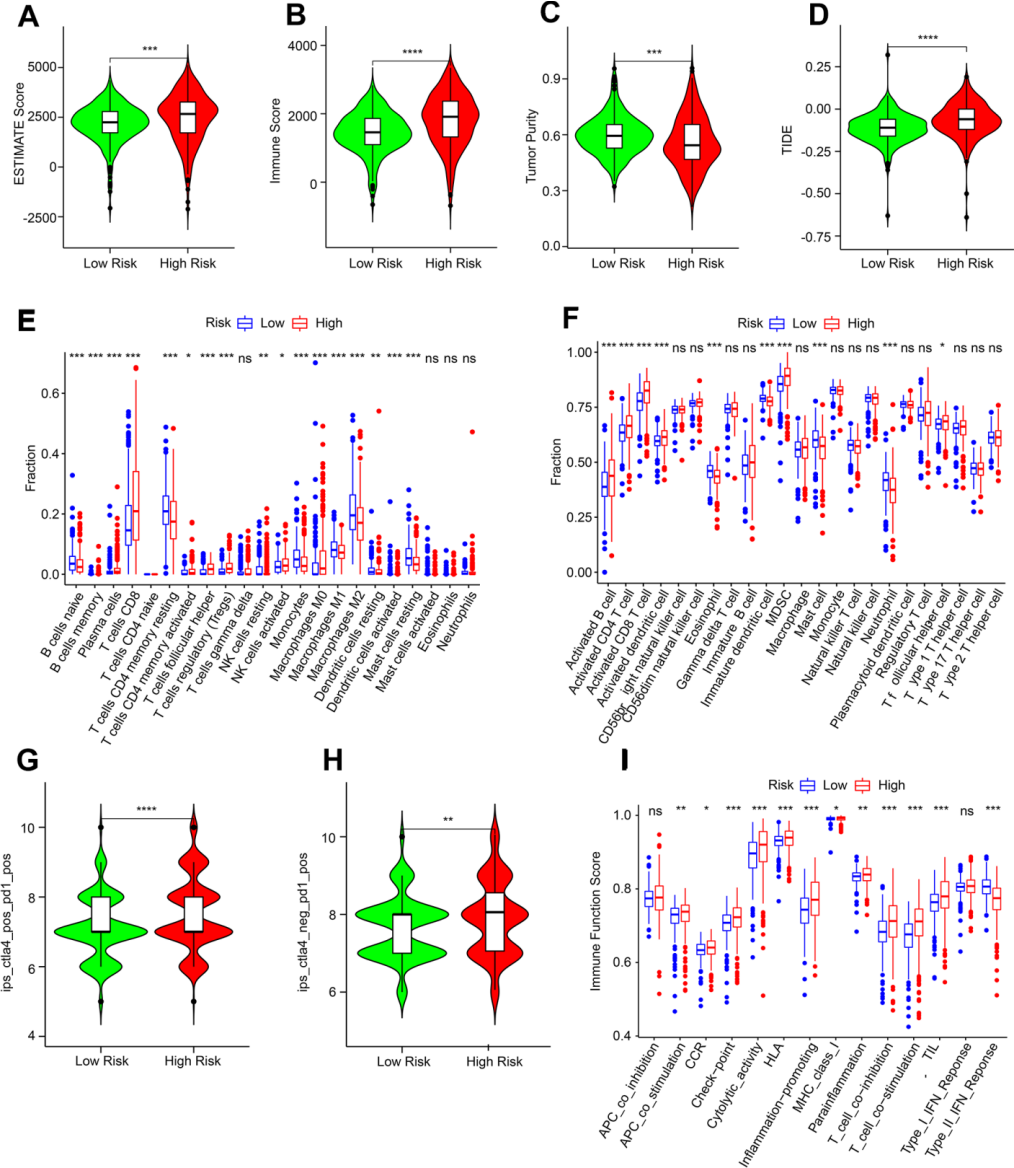
**Immune infiltration characterization of ARLs scores subgroups.** (**A**–**C**) ESTIMATE, immune scores and tumor purity. (**D**) TIDE score. (**E**, **F**) CIBERSORT and ssGSEA algorithm to estimate the immune cells fraction of ARLs score subgroups. (**G**, **H**) IPS score. (**I**) Immune function score.

The immune microenvironment landscape of patients by risk stratification was further evaluated. High-risk score patients had higher ESTIMATE level, immune score level and lower tumor purity level (Figure 8A–8C). Subsequent result showed lower TIDE scores could be observed in the low-risk score patients, indicating better outcomes of immunotherapy response for ccRCC (Figure 8D). CIBERSORT result indicated that the proportion of naïve B cells, monocytes, resting CD4^+^ memory T cells, resting NK cells, M2 macrophages, resting and activated dendritic cells were significantly higher for low-risk patients, while memory B cells, plasma cells, CD8^+^ T cells, TfH cells, M0 and M1 macrophages, Tregs and activated NK cells, were higher for high-risk score patients (Figure 8E). ssGSEA algorithm result indicated that the proportion of eosinophil, mast cell, immature dendritic cell and neutrophil were higher in the low-risk group, whereas the fraction of activated B, CD4^+^ T, CD8^+^ T and dendritic cells, as well as MDSC and TfH cell were higher in the high-risk group (Figure 8F).

Considering the remarkable difference in immune microenvironment by risk stratification, the response of immunotherapy was further evaluated. IPS results illustrated that high-risk score might lead to better response to immune checkpoint inhibitor-related therapy (Figure 8G, 8H). Immune function result showed that most of immune score were higher in patients with high-risk, whereas the type II IFN response level was lower (Figure 8I). The above result showed the ARLs risk model was closely associated with immune infiltration, and there may be differences in immunotherapy response in the ARL risk stratification.

### Drug sensitivity analysis of different risk groups

**Figure 9:**
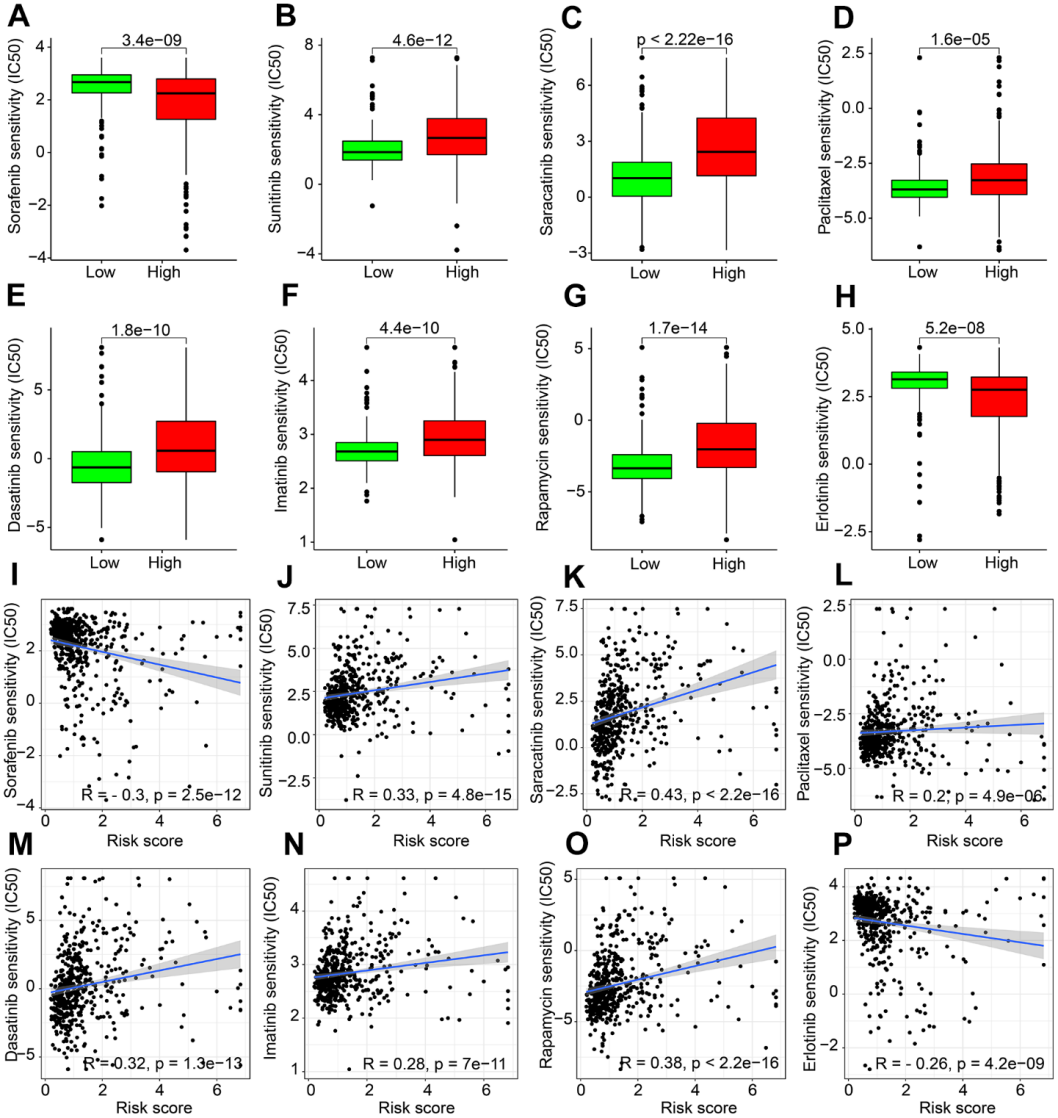
**Drug sensitivity exploration.** The distribution of IC50 in (**A**) Sorafenib. (**B**) Sunitinib. (**C**) Saracatinib. (**D**) Paclitaxel. (**E**) Dasatinib. (**F**) Imatinib. (**G**) Rapamycin. (**H**) Erlotinib. (**I**–**P**) Correlation analysis of ARLs prognostic signature and drug sensitivity (IC50).

Targeted drug therapy and chemotherapy has been considered as vital strategies in ccRCC clinical management. Thereafter, the sensitivity differences to several potential antineoplastic drugs by risk scoring stratification were evaluated. As shown in Figure 9A–9H, Sorafenib and Erlotinib had higher IC50 in the low-risk group; while high risk score patients had significantly higher IC50 of Sunitinib, Saracatinib, Paclitaxel, Dasatinib, Imatinib, and Rapamycin. Correlation analysis suggested that the risk score was negatively associated with IC50 of Sorafenib and Erlotinib, whereas positively associated with IC50 of Sunitinib, Dasatinib, Saracatinib, Imatinib, Paclitaxel and Rapamycin (Figure 9I–9P).

## DISCUSSION

In this study, a ARLs prognostic model of for ccRCC patients was established and its effectiveness was successfully verified. Among the five lncRNAs we screened, AC092611.2 and AC027601.2 were reported as prognostic markers in ccRCC [27, 28]. AL162586.1, ac103809.1 and AL133215.2 have not been reported. In RCC, many carcinogenic lncRNAs were overexpressed, while many tumors suppressor lncRNAs were down-regulated [29, 30]. A variety of lncRNAs have been screened to be critical for the progression of RCC and serve as markers of poor prognosis in patients [20, 31, 32]. In addition, the impact of some lncRNAs on the immune response of RCC has been suggested in the literature. LINC00973 was reported to increase the expression of siglec-15, which is a cancer cell surface antigen [33]. In terms of molecular mechanisms, the activation of multiple signaling pathways in RCC, including epithelial-mesenchymal transition process, hedgehog, PI3K/AKT and the VEGF signaling have been shown to be associated with lncRNA [34]. LncRNA has been reported to affect tumor development through anoikis process [35]. Our results further illustrate the role of lncRNAs in RCC and provide new research targets.

Our results of pathway enrichment analysis illustrated that our prognostic typing was closely correlated with immune-related pathways including adaptive immune response and phagocytosis. As an important part of anoikis process, ECM is associated with immunity in tumor development [36]. Traditionally, the ECM was thought to serve only as a scaffolding, but recently, its role in carcinogenesis has become increasingly clear. The ECM physical properties, such as ECM porosity, rigidity and insolubility can affect the biological functions of resident cells including the formation of immune microenvironment [36, 37]. There is already evidence that ECM rigidity strongly affects T-cell biological functions including activation, proliferation and differentiation [38]. In the tumor microenvironment, elevated hypoxia and metabolic stress could lead by poor diffusion of tumor ECM, leading to upregulation of TGF-β and VEGFA, which are commonly considered as immunosuppressive factors [39, 40]. ECM is also involved in tumor-related inflammatory responses, such as the polarization of tumor-associated macrophages (TAM), which makes macrophages biased toward M1 polarization and enhances the cytotoxic activity of macrophages against tumor cells [41]. Our data also showed a strong correlation between ECM-associated anoikis and immunity. The specific functions and underlying mechanisms of ECM-associated anoikis in tumor-related immunity need to be further explored.

Our results suggest a correlation between anoikis and response to immunotherapy. Although no association of anoikis with tumor immunotherapy has been reported so far, ECM has been reported to be involved in the antitumor immunotherapy process. During carcinogenesis process, the structure, physical properties and metabolism of ECM are highly dysregulated [42]. The ECM in tumoral cells is at least 1.5 times more rigid than the extracellular matrix in normal tissues [43]. On this basis, cell-ECM adhesion in tumor tissues is enhanced and cell-cell contact is disrupted, leading to tumor growth and metastasis [44]. The expression of PD-L1 is crucial to the immune escape process of tumor cells [45]. PD-L1 expression can be elevated through the regulatory mechanism of rigid ECM on actin, leading to immune system escape and tumor growth [46, 47]. Rigid ECM may also act as a physical barrier to T cell infiltration and localization; therefore, the anti-tumor immune behavior is disturbed [48]. In contrast, loose regions of glycoproteins and collagen in the ECM tend to promote T cell motility [49]. In addition, during T cell activation, an increase in HA binding capacity enhances T cells to roll on HA substrates, leading to better T cell migration and extravasation [50]. In addition, ECM can regulate DC maturation. Exposure to HA fragments can regulate the level of DC activation, thereby regulating the process of cancer antigen presentation [51]. At the same time, the density of tumor ECM can regulate the distribution of drugs and immune cell infiltration in tumors [52]. Based on the above findings, ECM has become one of the popular anticancer targets [53]. Targeting both collagen and hyaluronic acid in ECM has been reported [54]. Highly expressed collagen is associated with poor overall survival and affects response to chemotherapy, radiotherapy, and immunotherapy [55]. Drugs coupled with collagen-binding antibody fragments targeted to tumors with collagen-rich ECM resulted in a more durable antitumor effect in tumors [56]. A kind of hyaluronidase named PEGPH20, has been shown to successfully degrade HA in tumors and reshape tumor stroma through modulation of ECM components, thereby improving perfusion and drug delivery [57]. Further studies of ECM and anoikis have potential clinical applications.

Our study for sure has shortcomings. The analysis in this paper is correlation analysis without causal analysis, so the value of anoikis cannot be further analyzed. This paper lacks experimental data support, and further *in vitro* or *in vivo* experiments will better verify the conclusions of this paper. In addition, our results showed high-risk patients benefit more from immune-related therapy. However, we did not find any evidence of patients receiving immunotherapy in the public database. Stratified correlation analysis of real-world ccRCC immunotherapy response and risk stratification will have positive clinical significance. In conclusion, our data demonstrate the predictive value of anoikis-associated lncRNA prognostic models for patients with ccRCC.

## Supplementary Material

Supplementary Figure 1

Supplementary Tables
