# Supplementary material for: Validation and identification of anoikis-related lncRNA signatures for improving prognosis in clear cell renal cell carcinoma
Source: Aging (Albany NY). 2024 Feb 21;16(4):3915–33. doi: 10.18632/aging.205568 (PMC10929799; doi:10.18632/aging.205568)
Supplement: Supplementary Figure 1 [file aging-16-205568-s001.pdf]

SUPPLEMENTARY FIGURE

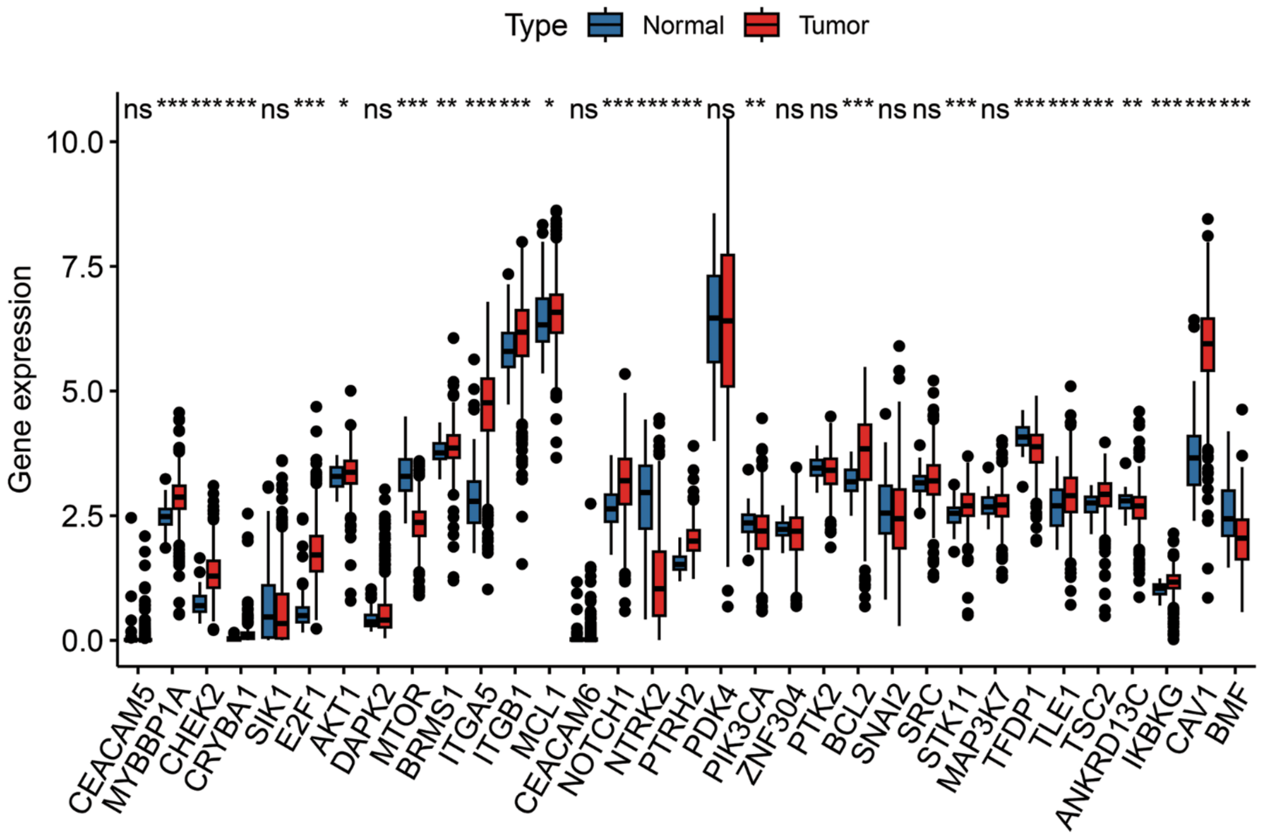

Supplementary Figure 1. The expression profiler of anoikis-related genes in normal and ccRCC samples.
