# Supplementary material for: Validation and identification of anoikis-related lncRNA signatures for improving prognosis in clear cell renal cell carcinoma
Source: Aging (Albany NY). 2024 Feb 21;16(4):3915–33. doi: 10.18632/aging.205568 (PMC10929799; doi:10.18632/aging.205568)
Supplement: Supplementary Tables [file aging-16-205568-s002.pdf]

SUPPLEMENTARY TABLES

Supplementary Table 1. The gene list of anoikis-related gene signature.

CEACAM5
MYBBP1A
CHEK2
CRYBA1
SIK1
TLE5
E2F1
AKT1
DAPK2
MTOR
BRMS1
ITGA5
ITGB1
MCL1
CEACAM6
NOTCH1
NTRK2
PTRH2
PDK4
PIK3CA
ZNF304
PTK2
BCL2
SNAI2
SRC
STK11
MAP3K7
TFDP1
TLE1
TSC2
ANKRD13C
IKBKG
CAV1
BMF

Supplementary Table 2. Identification of anoikis-related lncRNAs.

AL356599.1
AC012615.6
AL031714.1
AL139287.1
AC005034.5
AC124312.2
AL121832.2
AL731577.2
AL162586.1
LINC01023
AP001505.1
AL136084.3
AC138028.4
AC114730.3
AC011472.1
AC027601.2
NORAD
RUSC1-AS1
AC108449.2
AL022328.2
AC022150.2
AP001486.2
PAXIP1-AS2
OIP5-AS1
AC103809.1
Z68871.1
AC093278.2
RAP2C-AS1
AC023509.4
AC084018.1
AL022322.1
AC008735.2
AC107375.1
AL133215.2
AC108693.2
AC040169.1
HID1-AS1
AC073073.2
AL022328.1
AC109460.2
ZSCAN16-AS1
AL355803.1
CACTIN-AS1
AC092611.2
